# Compartmentalized cross-linked enzyme nano aggregates (*c*-CLE*n*As) toward pharmaceutical transformations†

**DOI:** 10.1039/d1ra04332c

**Published:** 2021-06-21

**Authors:** M. Teresa de Martino, Fabio Tonin, Victor R. L. J. Bloemendal, Ulf Hanefeld, Floris P. J. T. Rutjes, Jan C. M. van Hest

**Affiliations:** Department of Chemical Engineering & Chemistry, Institute for Complex Molecular Systems, Eindhoven University of Technology PO Box 513 5600 MB Eindhoven The Netherlands J.C.M.v.Hest@tue.nl; Department of Biotechnology, Delft University of Technology Van der Maasweg 9 2629 HZ Delft The Netherlands u.hanefeld@tudelft.nl; Institute for Molecules and Materials, Radboud University Heyendaalseweg 135 6525 AJ Nijmegen The Netherlands floris.rutjes@ru.nl

## Abstract

A new immobilization strategy using compartmentalized nanoreactors is herein reported for two biocatalytic processes: (1) *N*-acetylneuraminate lyase (NAL) is internalized in NAL-*c*-CLE*n*As and used in a continuous flow aldol condensation of *N*-acetyl-d-mannosamine with sodium pyruvate to *N*-acetylneuraminic acid; (2) two hydroxysteroid dehydrogenases (HSDH) 7α- and 7β-HSDH are incorporated in *c*-CLE*n*As and used in a two-step cascade batch synthesis of ursodeoxycholic acid (UDCA). The versatile use of *c*-CLE*n*A demonstrates that this immobilization methodology is a valuable addition to the toolbox of synthetic chemists.

## Abstract 2

A new immobilization strategy using compartmentalized nanoreactors is herein reported for two biocatalytic processes: (1) a continuous flow aldol reaction; (2) epimerization for the synthesis of ursodeoxycholic acid (UDCA).

## Graphical abstract

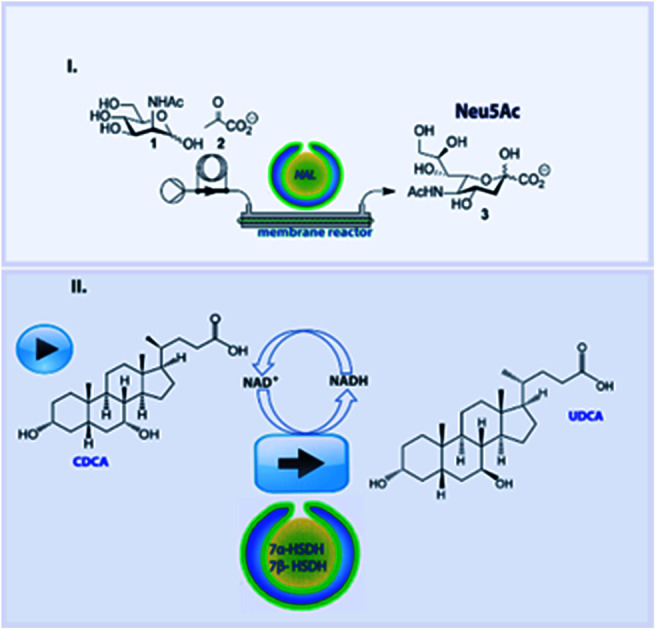

In the recent decade, there has been an ongoing search for effective implementation of enzymes in flow catalysis and multistep synthesis.^1–6^ The use of enzymes in flow can be highly beneficial as it allows combination of the stereo and substrate selectivity of the biocatalysts with excellent control in mass and heat transfer of the flow process.^7,8^ In addition, flow applications with enzymes allow for improved kinetic control, as reactions can be steered away from equilibrium to obtain higher conversions, which is difficult to achieve in batch reactions. All this makes flow catalysis a green and environmentally benign approach for pharmaceutically relevant transformations.^8–12^ However, to make these biocatalytic processes economically feasible, enzyme recycling is often required, which necessitates enzymes to be immobilized and retained in the flow reactor. Although many immobilization strategies have been developed, they are often hampered by a loss of activity as a result of the conjugation or adsorption method employed and/or of the reduced accessibility of the catalytic sites.^1^

To address this issue, recently, nanoreactors have been developed which can accommodate different catalytic species, allow effective substrate access and simplify workup operations.^13–16^ In our previous work, we created nanoreactors based on bowl-shaped poly(ethylene-glycol)-*b*-polystyrene (Fig. S1†) polymer vesicles, named stomatocytes, which could be loaded with enzymes in their nanocavity. Upon treatment with a crosslinking agent we obtained compartmentalized cross-linked enzyme *nano* aggregates (*c*-CLE*n*A) (Fig. S2†) which were shown to be beneficial for preserving the native enzymatic activity. In the case of combined-cross-linking of two enzymatic species, they resulted in enhanced activity.^17–19^ Additionally, *c*-CLE*n*As proved to be stably loaded with enzymes with minimal leaching during flow operations.^17^ Until now, the concept of *c*-CLE*n*As was demonstrated with robust model enzymes. In order to indicate the added value for the fine chemical and pharmaceutical industry, in this work we have investigated two applications for *c*-CLE*n*As as efficient biocatalytic nanoreactors for a continuous flow aldol condensation and a one-pot biocatalytic two-step reaction (Fig. 1) toward useful pharmaceutical intermediates.

**Fig. 1:**
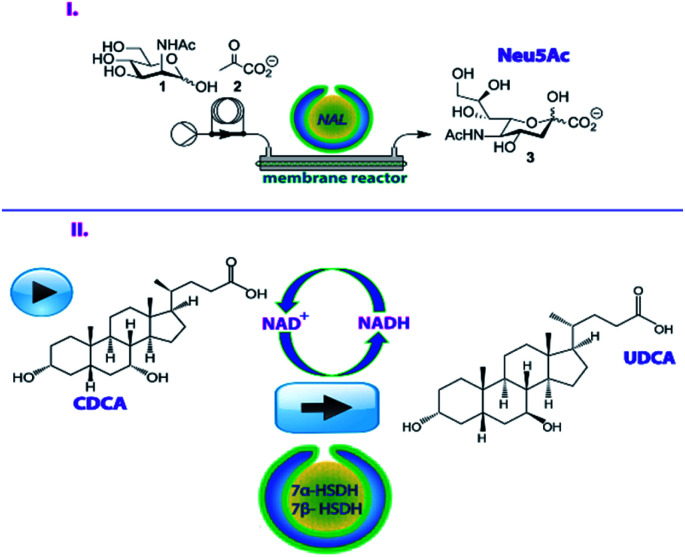
Compartmentalized-cross-linked enzyme *nano*-aggregates *c*-CLE*n*As for the execution of enzymatic transformations with pharmaceutical relevance (I) NAL-*c*-CLE*n*As for a continuous flow synthesis of Neu5Ac; (II) Combined 7α/7β-HSDH *c*-CLE*n*As for a one-pot cascade reaction to UDCA.

For the first example, we focused on *N*-acetylneuraminate lyase (NAL, EC 4.1.1.3) which is a well-studied enzyme for the preparation of neuraminic acid derivatives (Neu5Ac).^20,21^ Moreover, NAL demonstrates high substrate variability and enzymatic stability (Fig. 1I). Neu5Ac and its derivatives have a high relevance from a pharmacological point of view since they are involved in a range of physiological processes.^20^ Derivatives of Neu5Ac have for example been used to inhibit the neuraminidases of influenza viruses A and B in a clinical setting and they are known to prevent avian influenza.^22,23^ NAL has already been encapsulated in cross-linked enzyme aggregates (CLEA)^24^ and cross-linked inclusion bodies (CLIB)^25,26^ which were both effective in retaining enzymatic activity but were challenging to prepare. In other studies, NAL has been immobilized on immobeads, but this process required considerable quantities of enzymes to produce reasonable amounts of Neu5Ac.^20^ We hypothesized that using *c*-CLE*n*As as a novel immobilization strategy for NAL would allow the effective implementation of this nanoreactor in a flow process with more economical use of the enzyme.

Secondly, the two enzymes 7α- and 7β-hydroxysteroid dehydrogenase (7α-HDSH and 7β-HSDH) were encapsulated in stomatocyte nanoreactors to enable the cascade reaction for the synthesis of ursodeoxycholic acid (UDCA) (Fig. 1II).^27^ The chemical transformation to UDCA, starting from chenodeoxycholic acid (CDCA), can be performed by 7α-HSDH and 7β-HSDH that are respectively NAD^+^ and NADH dependent.^27^ UDCA is an important secondary bile acid that is used as a pharmaceutical product in the clinic to improve liver function and solubilize cholesterol gallstones.^28,29^ Traditionally, the non-enzymatic route for UDCA production involves many reaction steps, including the use of hazardous and toxic solvents and the final product is only recovered in a low yield (∼30%).^30^ To make the synthesis more effective an enzymatic route has been developed, starting from cholic acid (CA), the cheapest bile acid available. The route entails C12 dehydroxylation, followed by 7-OH epimerization. For the latter process 7α-HSDH and 7β-HSDH work in a cascade fashion. The 7α-OH group of CDCA is oxidized by 7α-HSDH, with the concomitant reduction of NAD^+^ to NADH. The obtained compound (7-oxo-lithocholic acid, 7-oxo-LCA) is subsequently reduced to the final product (UDCA) by 7β-HSDH that utilizes the NADH produced in the first reaction. Notably, this epimerization reaction is carried out in a redox-neutral manner, where the equilibrium between CDCA and UDCA is solely thermodynamically determined. Optimization of this enzymatic route can be achieved by an effective immobilization of these two complementary enzymes, allowing for an easy separation from the final product, without hampering their activity and without creating diffusional barriers for the substrates and cofactors. With this in mind, we developed the co-immobilization of 7α-HSDH and 7β-HSDH in the same *c*-CLE*n*A nanoreactors, hypothesizing that the close proximity of the two species in the nanosized cavity of the stomatocytes would bring additional stability and high reactivity to this cascade.

In previous reports it was described that NAL is a selective but rather slow converting enzyme. As a result, to make continuous flow production of Neu5Ac feasible, the loading of NAL was an important design aspect to take into consideration.^20^ Therefore, the preparation of NAL-*c*-CLE*n*As required optimization from the previously described procedure.^17^ In the first instance it was attempted to create stomatocyte samples with a loading efficiency of ∼30% (loading efficiency calculated by Bradford assay, corresponding to 3 mg mL^−1^ of enzyme encapsulated starting from 10 mg mL^−1^ NAL solution). The enzyme was subsequently cross-linked with either genipin (1 wt%) or glutaraldehyde (100–300 mM).^31^ However, these formulations showed severe problems. The formation of aggregates at the bottom of the solution was observed in the case of glutaraldehyde cross-linking, which was accompanied by a complete deactivation of the enzyme. As observed before, the fast reaction with glutaraldehyde resulted in denaturation of the enzymes. Furthermore, the high enzyme loading led to cross-linking between particles, causing precipitation. When a solution (1 wt%) of the milder cross-linking agent genipin was used for the NAL-*c*-CLE*n*A preparation this latter problem was resolved. However, also in this case enzyme activity was negligible. This was not observed when using *ca.* 14% loaded samples (1.4 mg NAL loaded in 500 μL of 10 mg mL^−1^ stomatocytes). Enzyme activity was preserved and no aggregation was observed. This NAL-*c*-CLE*n*A was subsequently used for the flow experiments (Table S1†).

Several solutions of 14% loaded NAL-*c*-CLE*n*As were tested in the continuous flow aldol reaction of a stock solution of *N*-acetyl-d-mannosamine (1) and sodium pyruvate (2) using a membrane reactor (Table S1†). For ensuring a NAL loading of at least 20 mg in the membrane reactor, 10 mL samples (10 mg mL^−1^) were concentrated to 3 mL and the final solution was loaded in the membrane. The catalytic activity was investigated by varying flow rates, temperature, and also the type of membrane (Table S1†). When 500 mM of (1) and 100 mM of (2) were fed at 35 °C over a modified poly(ether sulfone) (mPES) membrane (10 kDa) at 20 μL min^−1^ 69% yield of Neu5Ac was observed. At higher temperatures (50 °C), the activity was decreased to 50%. Increasing the flow rate resulted in a lower conversion, as expected. However, for the highest flow rate tested (100 μL min^−1^) the conversion was still 35%.

The obtained conversions were comparable to previous results reported.^24^ Still, the use of NAL-*c*-CLE*n*A effectively decreased enzyme loading by a factor of ∼3, thus increasing the turnover number of NAL. Furthermore, the stability of NAL-*c*-CLE*n*As in flow was also object of our investigation (Fig. 2).

**Fig. 2:**
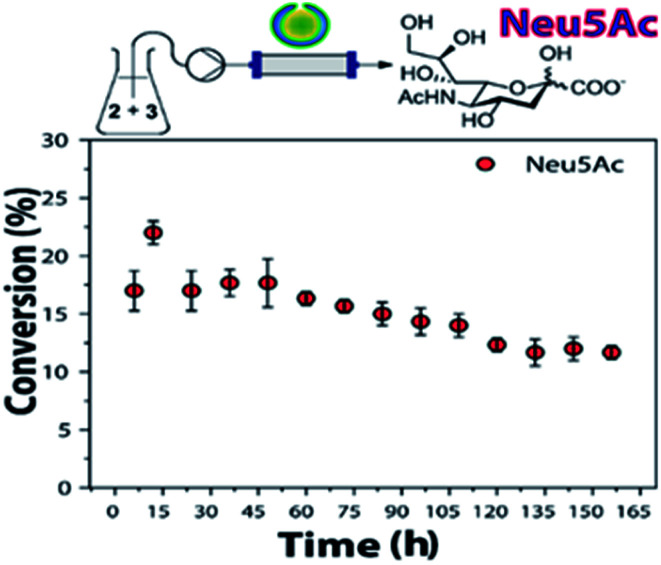
Stability test of NAL-*c*-CLE*n*A in a continuous flow setup. Conditions: ManNAc (2, 500 mM), sodium pyruvate (3, 100 mM), H_2_O, NAL-*c*-CLE*n*A (3 mL of 0.34 g mL^−1^, containing 18 mg of NAL). 25 μL min^−1^, 35 °C. Conversions determined by ^1^H NMR analysis of the crude reaction mixture (Fig. S3†).

For the stability experiments, the flow setup was based on a hollow fiber reactor with a membrane of mixed cellulose ether (ME) with 0.1 μm pore size. This membrane was chosen as it would allow for the retention of the polymeric nanovesicles in the tubular reactor but not of the enzymes in case of leaching. Based on earlier results with *c*-CLE*n*As leaching could be ruled out and with this experiment we could therefore specifically focus on enzyme deactivation.^17^

The conditions were set to ensure a conversion below 50%, in order to have a more accurate insight in the change of conversion over time as a function of deactivation. Gratifyingly, when NAL-*c*-CLE*n*As were continuously tested in the flow reactor for a week, Neu5Ac formation was still observed. The use of NAL immobilized-beads resulted in ∼33% loss in activity in a previous report;^20^ we proved that our NAL-*c*-CLE*n*As demonstrated similar stability (∼31% loss after the same period).

The *c*-CLE*n*A system cannot only be useful for application in flow systems, but the nano-assembly method also allows to bring enzymes together that operate in a cascade fashion. This was demonstrated with the enzymatic redox-neutral epimerization of CDCA. Recently, this reaction has been carried out by employing two enzymes (7α-HSDH and 7β-HSDH).^27^ Here a batch reaction is highly favoured over the flow approach. In flow the cofactor would be flushed away and the reaction could then not be performed in a redox neutral manner anymore.

The enzymes were produced and purified according to literature.^27^ To probe for proximity effects, different nanoreactors were prepared by encapsulating the two enzymatic species either separately or together. Firstly, the two enzymes were encapsulated separately, leading to the construction of 7α-HSDH-*c*-CLE*n*As and 7β-HSDH-*c*-CLE*n*As, both with a loading efficiency of 25% (2 mg of enzyme loaded in 500 μL of 10 mg mL^−1^ sample). The optimal amount of genipin needed for the cross-linking of the enzymes was established by analysing the residual specific activity of the enzymes after encapsulation and cross-linking. Similarly to the NAL-*c*-CLE*n*As, using glutaraldehyde as cross-linker deactivated both enzymes significantly suggesting that using a faster bifunctional cross-linker might not be suitable for these particular enzymes. In contrast to the previous example with NAL none of the *c*-CLE*n*As showed the formation of clustering or sedimentation. When a genipin solution at 1 %wt was used to cross-link the samples the activity of both enzymes was conserved. Notably, batch-to-batch variations were observed using different amounts of genipin (Table S2†). Next, the co-encapsulation of the two enzymes in a single nanoreactor was investigated. Ideally, co-encapsulation is advantageous since it reduces the diffusion limitations of the reagents and cofactors (CDCA/7-oxo-LCA and NAD^+^/NADH). The encapsulation and cross-linking processes were carried out using the same procedure as for the single enzyme encapsulation; therefore, to 25% loaded 7α/7β-HSDH stomatocyte samples (2 mg of total enzyme mixture loaded in the sample), 1 wt% genipin was added to afford 7α/7β-HSDH *c*-CLE*n*As. A sample of co-encapsulated enzymes without cross-linking (7α/7β-HSDH stomatocytes, 25% loading efficiency) was used as control to evaluate the effect of the cross-linking step on the enzymatic activities. Notably, stomatocytes with non-crosslinked enzymes and *c*-CLE*n*A showed similar activities. This indicates that, the enzymes conserved their activity when a milder cross-linker as genipin is used. To establish the applicability of the newly obtained nanoreactors for the epimerization of CDCA into UDCA, a series of bioconversions was performed (Fig. 3).

**Fig. 3:**
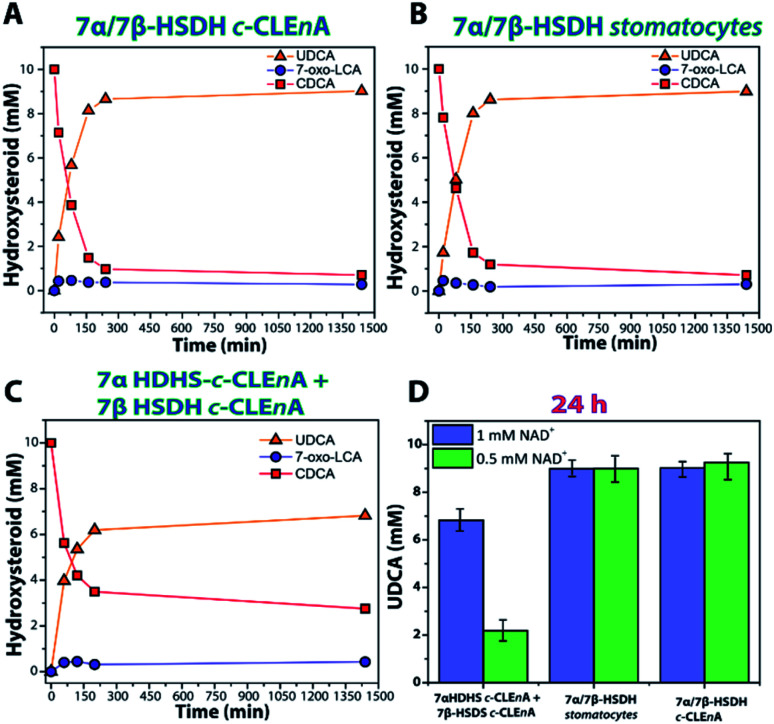
Bioconversion of 10 mM CDCA into UDCA with 1 mM of NAD^+^ by using (A) co-encapsulated 7α/7β-HSDH *c*-CLE*n*A (B) co-encapsulated 7α/7β-HSDH stomatocytes (C) 7α + 7β-HSDH in separate *c*-CLE*n*As. (D) Bar plot reporting the amount of UDCA (mM) formed after 24 h reaction with 0.5 mM and 1 mM of NAD^+^. 7-Oxo-LCA is the intermediate product 7-oxo-lithocholic acid.

When the cascade reaction was performed using the co-encapsulated enzymes 7α and 7β-HSDH in stomatocytes or in *c*-CLE*n*As (Fig. 3A and B) no significant differences between the two nanoreactors were observed. Using a catalyst loading of 200–300 μg (equivalent to 1.5 U and 0.9 U of 7α- and 7β-HSDH activity, respectively) and 1 mM of NAD^+^, 8.6 mM (conversion 86%) and 8.7 mM (conversion 87%) of UDCA were obtained after 200 min using 7α/7β-HSDH stomatocytes and 7α/7β-HSDH *c*-CLE*n*A, respectively. In comparison to literature in which the free enzymes were used, the reaction with *c*-CLE*n*A showed comparable rates and conversion values.^30^ In addition, when a catalyst loading of 20–30 μg (equivalent to 0.15 U and 0.09 U of 7α- and 7β-HSDH activity, respectively) was used, 9.0 mM of UDCA was obtained, (90% conversion) after 24 h using both kinds of preparations. Similar conversions were obtained when 0.5 mM NAD^+^ was used (Fig. 3D). On the other hand, when bioconversions were performed at similar conditions using separately encapsulated 7α + 7β-HSDH *c*-CLE*n*As (1 U_TOT_ of 7α HSDH *c*-CLE*n*A (2.6 μg) and 0.8 U_TOT_ of 7β-HSDH *c*-CLE*n*A (133 μg)) lower activities were observed (Fig. 3C). When 1 mM of NAD^+^ was used, 6.2 mM (62% conversion) and 6.7 mM (67% conversion) of UDCA were obtained after 200 min and 24 h, respectively. Additionally, when 0.5 mM of NAD^+^ was used with separately encapsulated 7α + 7β-HSDH *c*-CLE*n*As, only 2.2 mM of UDCA (22% conversion) was obtained after 24 h (Fig. 3D).

Our experiments suggest that having the enzymes encapsulated in two different compartments poses an additional barrier to the substrate/co-factor diffusion to activate the second step of the cascade, impacting the final conversion which is considerably lower compared to the free enzyme cascade.

This is especially visible when the co-factor concentration is lowered, and the final conversion is 22%. However, this is not observed with the 7α/7β-HSDH *c*-CLE*n*A and stomatocytes in which the bioconversions show good agreement with the cascade reaction performed with the 7α and 7β-HSDH as free enzymes. In these systems there is no diffusional constraint for the second enzymatic conversion. This demonstrates that the co-encapsulation in stomatocytes allows the cascade reaction to be performed without negative effects on conversion, but with the potential benefit of easy catalyst recovery.

In summary, *c*-CLE*n*As were implemented for the *N*-acetylneuraminic acid lyase mediated production of Neu5Ac in flow, and for the bioconversion of CDCA to UDCA *via* 7α-HSDH and 7β-HSDH. Both nanoreactor systems showed to be inactive when cross-linked using glutaraldehyde but gratifyingly retained enzymatic activity upon controlled crosslinking with genipin. For the *N*-acetylneuraminic acid lyase synthesis, the NAL-*c*-CLE*n*As were loaded in a hollow fiber membrane flow reactor. After optimizing the reaction conditions by using a modified polyethersulfone reactor and low flow rates a conversion of 69% to Neu5Ac was achieved. Although this yield did not outrank previous results with the immobead-NAL, it effectively decreased enzyme loading by a factor of three, thus increasing the turnover number of the neuraminic acid lyase. The stability of the NAL-*c*-CLE*n*As was further demonstrated in a continuous experiment for 168 hours with a moderate loss in their activity. Regarding the cascade to UDCA, the activity of the two enzymes was preserved after cross-linking the two enzymes in the same stomatocytes with genipin, and no significant difference was observed between the not crosslinked enzyme-loaded stomatocytes and the *c*-CLE*n*A. However, the combined *c*-CLE*n*A proved to be more active than when the two enzymes were compartmentalized in separated *c*-CLE*n*As, especially at lower concentrations of the cofactor NADH. This demonstrates that two pharmaceutically relevant transformations can be effectively performed with the outlined systems, indicating that this technology can be broadly applied in biocatalysis for drug synthesis.

## Conflicts of interest

There are no conflicts to declare.

## Supplementary Material

RA-011-D1RA04332C-s001
