# Supplementary material for: Compartmentalized cross-linked enzyme nano aggregates (c-CLEnAs) toward pharmaceutical transformations
Source: RSC Adv. 2021 Jun 21;11(35):21857–61. doi: 10.1039/d1ra04332c (PMC9034143; doi:10.1039/d1ra04332c)
Supplement: RA-011-D1RA04332C-s001 [file RA-011-D1RA04332C-s001.pdf]

Electronic Supplementary Material (ESI) for RSC Advances.
This journal is © The Royal Society of Chemistry 2021

Electronic Support Information for:

"Compartmentalized cross-linked enzyme *nano* aggregates (c-CLE*n*As) toward pharmaceutical transformations"

Authors: M. Teresa de Martino, Fabio Tonin, Victor R.L.J. Bloemendal, Ulf Hanefeld, Floris P.J.T. Rutjes and Jan C.M. van Hest

Contents

1.1. Materials and Methods 1
1.2. Experimental procedures 3
1.2.1. Synthesis of poly(ethylene glycol)$_{44}$-polystyrene (PEG$_{44}$-*b*-PS$_{200}$) block copolymer 3
1.2.2. General procedure for polymersome preparation 4
1.2.5. General procedure for the formation of compartmentalized cross-linked enzyme nano aggregates (*c*-CLE*n*A) with genipin 5
1.2.6. Quantification of enzyme loading 5
References 6
1.3. Supplementary figures and table 6

1.1. Materials and Methods

All chemicals and enzymes were purchased from Sigma-Aldrich and used as received unless otherwise stated. For the synthesis of the block copolymer, CuBr was mixed in acetic acid for 3 h and dried in vacuum. Ultra-pure MilliQ water (Labconco Water Pro PS purification system) was used for the self-assembly of polymersomes and dialysis. The dialysis membranes (MWCO 12-14 kDa Spectra/Por®), Amicon Ultra- 0.5 mL centrifugal filter Unit 3 kDa (Millipore), and Ultrafree-MC centrifugal filters (with 0.1 and 0.22 µm pore size) (Millipore) were used to remove the excess of enzyme after encapsulation, and to wash the nanoreactors after the flow experiments. *N*-acetylneuraminate lyase - crystalline (EC 4.1.3.3) was supplied by Biosynth Carbosynth ®, 7α-HSDH and 7β-HSDH were recombinantly produced and purifiedas decribed earlier. The glutaraldehyde solution (25% w/w in H_2O) was purchased from Sigma Aldrich and solutions at different concentrations were prepared for the *c*-CLE*n*A formation. Genipin (≥98% (HPLC) in powder) was also purchased from Sigma Aldrich and solutions at different concentrations were prepared in MilliQ water.

Proton nuclear magnetic resonance (^{1}H NMR): ^{1}H NMR spectra were recorded on a Varian Inova 400 spectrometer with $CDCl_3$ as a solvent and TMS as internal standard. ^{1}H NMR spectra were used to determine the molecular weight of the synthesized copolymers.

Gel permeation chromatography (GPC): The dispersity (Đ) of the copolymers was determined using a Shimadzu Prominence GPC system equipped with a PL gel 5 µm mixed D column (Polymer Laboratories) and differential refractive index and UV (254 nm) detectors. THF was used as an eluent at a flow rate of 1 mL min^{-1}.

Size Exclusion Chromatography (SEC): For an efficient separation of the stomatocytes from the unencapsulated enzymes, a Shimadzu Prominence SEC system equipped with a Superose™ 6 column and a UV detector (220 nm) was used. The separation was performed using filtered PBS buffer at 0.8 mL min^{-1}.

Transmission electron microscopy (TEM): TEM images were recorded using a FEI Tecnai 20 (type Sphera) at 200 kV. 5 µL sample was dropped on top of a carbon-coated copper grid *(*200 mesh, EM science*)*, and the desalted samples were left to dry at room temperature overnight.

High Performance Liquid Chromatography (HPLC): HPLC analyses were performed on a Shimadzu apparatus equipped with an LC20AT pump and an ELSD-LTII detector and fitted with an XTerra RP C18 column (length/internal diameter 150/4.6 mm, pore size 5 µm) under the following conditions: eluent $H_2O/CH_3CN/TFA$ (65/35/1), flux 1.0 mL/min.

NAL- *c*-CLE*n*A Activity in Flow: Multiple NAL-*c*-CLE*n*As (10 mg mL^{-1}) containing ca 1.4 mg of NAL were mixed together to reach a final amount of ca 20 mg NAL. The final solution was adjusted to 3 mL via spin filtration (0.1 µm, 3000rpm) for 10 mins. The aqueous suspension was vortexed and loaded into a syringe (1 mL), after which it was coupled directly to the side-inlet of the flow reactor. Using a syringe pump, the solution was slowly added to the reactor (≤ 0.5 $mLmin^{-1}$), effectively eluting excess aqueous solvent. This procedure was repeated 3 × after which the reactor was sealed before the catalysis experiments. After the indicated time intervals (Table S1) the reaction mixture was separately collected for 10 min (3 ×). The samples were concentrated *in vacuo* and the conversion determined using ^1H NMR in D_2O.

NAL- *c*-CLE*n*A stability test in a membrane in-flow reactor: The Microkros ® reactor was loaded with *c*-CLE*n*A enzyme dispersion (containing up to 18 mg NAL) according to the aforementioned procedure. An HPLC pump was loaded with a solution of ManNAc (**1**, 500 mM) and sodium pyruvate (**2**, 100 mM) and the pH was adjusted to 7.0 using 1 M aqueous NaOH or HCl. The Microkros reactor was connected directly to the HPLC pump. The reactor was submerged in a 35 °C water bath for 10 minutes before starting the experiment. The flow rate was set to 25 $\mu Lmin^{-1}$, and the substrate solution was continuously pumped through the reactor. After the indicated time intervals, the reaction mixture was separately collected for 10 min (3 ×). The samples were concentrated *in vacuo* and the conversions were determined using ^1H NMR in D_2O.

Enzymatic assay: the enzymatic activity of 7α-HSDH and 7β-HSDH (on purified enzyme, encapsulated and co-encapsulated) was determined at room temperature (25 °C) using 2.0 mM CDCA or UDCA respectively, 1.0 mM NAD^+, in 50 mM KPi buffer and 10% methanol (v/v), pH 8.0. The 7α-HSDH activity was measured using the conversion of CDCA into the intermediate 7-oxo-LCA. The activity of 7β-HSDH was measured using the reverse reaction from UDCA to 7-oxo-LCA (as the intermediate compound 7-oxo-LCA is not commercially available). The extinction coefficients of NADH, at 340 nm is 6.220 $M^{-1}\cdot cm^{-1}$. One unit (U) was defined as the amount of enzyme producing 1 µmol of product per minute at 25 °C and at pH 8.0. Blank measurements were performed in the absence of CDCA, NAD^+ and enzyme. Results are reported in Table S2.

Epimerization of CDCA to UDCA with separately encapsulated enzymes (7α-HSDH -*c*-CLE*n*A and 7β-HSDH -*c*-CLE*n*A): All conversions were carried out (triplicates) employing 1 U_{TOT} of 7α-HSDH *c*-CLEnA (15 µL of a 10x diluted stock, 2.6 µg) and 0.8 U_{TOT} of 7β-HSDH *c*-CLEnA (333 µL, 133 µg) on 10 mM CDCA, using 1 or 0.5 mM NAD^+. As a

general procedure, 1 mL of reaction mixture containing 10% MeOH and 50 mM of KPi buffer, pH 8.0 was incubated at 25 °C on a rotatory wheel. At fixed times of incubation 50 µL of reaction mixture were diluted with 200 µL of mobile phase centrifuged in order to separate the nanoreactors from the mixture (14000 rpm, 2 min). The supernatant was filtered (syringe filter 0.2 µm) and 10 µL of these preparations were analyzed by HPLC.

Epimerization of CDCA to UDCA with co-encapsulated enzymes (7α/7β-HSDH stomatocytes and 7α/7β-HSDH -*c*-CLE*n*A): All conversions were carried out (duplicates) employing different amounts of 7α/7β-HSDH *c*-CLE*n*A (300 µL of different dilution, Table S3) on 10 mM CDCA, using 1 or 0.5 mM NAD^+. As a general procedure, 1 mL of reaction mixture containing 10% MeOH and 50 mM of KPi buffer, pH 8.0 was incubated at 25 °C on a rotatory wheel. At fixed times of incubation 50 µL of reaction mixture were diluted with 200 µL of mobile phase and centrifuged in order to separate the nanoreactors from the mixture (14000 rpm, 2 min). The supernatant was filtered (syringe filter 0.2 µm) and 10 µL of these preparations were analyzed by HPLC.

Bradford assay: The Bradford method was used to quantify the enzyme loading. Pierce™ Coomassie Plus (Bradford) assay kit was used as described in the protocol of the assay, Table S1 reports the amount of enzyme used in all the experiments.

1.2. Experimental procedures

1.2.1. Synthesis of poly(ethylene glycol)$_{44}$-polystyrene (PEG$_{44}$-*b*-PS$_{200}$) block copolymer

PEG-*b*-PS was synthesized using atom-transfer controlled radical polymerization (ATRP), according to previously reported literature procedures. For the macro initiator synthesis, poly(ethylene glycol) methyl ether (5.0 g, 2.5 mmol), was twice dried by co-evaporation with toluene. In a flame-dried Schlenk tube, the poly(ethylene glycol) methyl ether was then dissolved in dry THF (2.0 mL) and triethylamine (1.04 mL, 7.5 mmol) was added to the solution. The Schlenk tube was placed on an ice bath, followed by the dropwise addition of α-bromoisobutyryl bromide (616 µL, 5.0 mmol) while stirring. The solution was then stirred for a further 24 h, while slowly warming to room temperature, to form a white solid due to the amine salt in the colorless solution. The amine salt was filtered off and the solution was concentrated in vacuum. The precipitation of macro-initiator poly(ethylene glycol)$_{44}$ methyl ether 2-bromoisobutyrate was induced by ice-cold diethyl ether.

For the PEG$_{44}$-*b*-PS$_{200}$ synthesis, copper bromide (CuBr) (45.0 mg, 0.32 mmol) was first added to a flame dried Schlenk tube equipped with a stirring bar under argon atmosphere. The Schlenk tube was sealed with a septum, and evacuated for 15 min, after which argon was filled back into the flask. PMDETA (66.0 µL, 0.32 mmol) was dissolved in toluene (0.5 mL) and added to the CuBr powder. The mixture was left stirring for 15 min with argon for oxygen removal. Poly(ethylene glycol) macroinitiator (215 mg, 0.10 mmol), dissolved in toluene (1 mL), was added into the Schlenk tube. The solution was degassed for 15 min while cooling in an ice bath. Distilled styrene (5.0 ml, 43.6 mmol) was added to the reaction mixture. The mixture was then degassed and the Schlenk tube was inserted into a preheated 70 ^{o}C oil bath, overnight. At the end of the reaction, dichloromethane (DCM) (75 mL) was added to the polymer solution and the mixture was filtered over an alumina column to remove the CuBr. The final solution was then concentrated and the polymer was precipitated in cold methanol, filtered, and

dried overnight in vacuum. The amphiphilic polymer obtained, PEG_{44}-*b*-PS_{200} had a number average molecular weight (M_n) of 29.1 kg mol^{-1} and Đ= 1.08.

1.2.2. General procedure for polymersome preparation

The polymersomes were self-assembled using a slightly modified variation of a previously reported solvent switch method.

In short, 20.0 mg synthesized PEG_{44}-*b*-PS_{200} polymer was dissolved in a 2 mL mixture of THF: dioxane (4:1 v/v), to which 1.0 mL MilliQ was added via a syringe pump with a flow rate of 1.0 mL h^{-1}, resulting in the formation of a cloudy solution. The assembly was performed inside a 5.0 mL vial which contained a magnetic stirring bar and which was capped with a septum. The cloudy solution was then dialyzed against MilliQ water for 24 h, with the MilliQ frequently refreshed.

1.2.3. General procedure for stomatocyte preparation and enzyme loading

Stomatocyte nanoreactors were prepared using the previously reported solvent addition methodology.[1] 300 µL THF:dioxane solution (4:1 v/v) was added via syringe pump at a rate of 300 µL h^{-1} to 500 µL of the previously prepared polymersome solution (10.0 mg mL^{-1}), while continuously stirring. The organic mixture was removed from the polymeric solution using spin filtration (20 mins, 13523 rcf) which was repeated two times using Amicon 3 kDa filters). The polymersomes were re-suspended to their initial concentration by adding MilliQ water. At the end of this process, opened neck stomatocytes were formed which were used for enzyme entrapment.

Next, 1 mL of a 10 mg mL^{-1} NAL solution in 50 mM sodium phosphate buffer (50 mM pH 7.5), was added to the stomatocytes and mixed vigorously at 7000 rpm for 30 mins. To narrow the neck of the stomatocytes 150 µL THF: dioxane (4 : 1 v/v) at 150 µL h^{-1} flow rate was added to the solution. To remove the THF, samples were purified using spin filtration (15 mins, 13523 rcf) two times with Amicon 3 kDa filters. To remove non-encapsulated enzymes, stomatocytes were purified from the solution mixture using size exclusion chromatography (SEC). After SEC, the stomatocytes were concentrated again to a final volume of 500 µL (10 mg mL^{-1}). The same procedure was used for the 7α-HSDH and 7β-HSDH stomatocytes, using 8 mg mL^{-1} of 7α-HSDH and 8 mg mL^{-1} of 7β-HSDH or a solution of [7α-HSDH +7β-HSDH]=8 mg mL^{-1} with both enzymes. In the case of 7α-HSDH and 7β-HSDH, the samples were re-dispersed in PKi (50mM pH = 8).

1.2.4. General procedure for the formation of compartmentalized cross-linked enzyme nano aggregates (c-CLEnA) with glutaraldehyde

Having ensured complete removal of free enzyme from the previously prepared stomatocyte nanoreactors, glutaraldehyde (100 µL, at different concentrations varying between 100 mM and 300 mM) was slowly added, at a rate of 100 µL h^{-1}, to a 500 µL solution of enzyme loaded stomatocytes (10 mg mL^{-1}) while stirring. In the case of NAL samples, the cross-linking reaction was quenched with 1mL of sodium phosphate buffer (1M, pH = 7.5) solution, and in the case of 7α-HSDH and 7β-HSDH, the reaction was quenched with 1mL of PKi (50 mM pH = 8). To remove the excess of buffer and cross-linker glutaraldehyde, all resulting *c*-CLE*n*As were concentrated via spin filtration (15 mins, 13523 rcf) two times with Amicon 3 kDa filters and then were re-dispersed in sodium

phosphate buffer (50 mM, pH = 7.4). In the case of 7α-HSDH and 7β-HSDH, the *c*-CLE*n*A was re-dispersed in PKi (50mM pH = 8).

1.2.5. General procedure for the formation of compartmentalized cross-linked enzyme nano aggregates (*c*-CLE*n*A) with genipin

500 μL of genipin solution (1wt%-1.6wt%-0.75wt%) was added to 500 μL of stomatocyte sample (10 mg mL^{-1}) in an Eppendorf tube. The solutions were kept for 24h at RT under gentle stirring.

The optimal procedure for NAL *c*-CLE*n*A formation, employed a dispersion of 500 μL of NAL-stomatocytes (10 mg mL^{-1}) with a 14% loading efficiency (EE%) (corresponding to 1.4 mg of NAL encapsulated in 500 μL sample, determined by Bradford), which was mixed with 500 μL genipin 1wt%.

The optimal procedure for the formation of 7α-HSDH and 7β-HSD- *c*-CLE*n*As employed 500 μL stomatocytes (10 mg mL^{-1}) with a 25% loading efficiency (2 mg of enzyme loaded in 500 μL sample, determined by Bradford) of either 7α- HSDH or 7β-HSDH, which were mixed with 500 μL of genipin solution (1wt%). The co-encapsulated sample was prepared in the same way starting from 500 μL of 7α/7β-HSDH stomatocytes (10 mg mL^{-1}) with a loading efficiency of 25% (2 mg total enzymes loaded in the 500 μL sample, determined by Bradford).

After 24h genipin was removed from the solution, by using 10 kDa filters in a centrifuge at 12000 rpm for 15 min, the final concentration was adjusted to 10mg mL^{-1} with sodium phosphate buffer for the NAL sample or with 50mM PKi buffer (pH 8) for the 7α- HSDH and 7β-HSDH samples.

1.2.6. Quantification of enzyme loading

The Bradford assay was used to quantify the amount of enzyme loaded in the stomatocytes and in the *c*-CLE*n*As. All the samples were treated with CH_2Cl_2 to completely remove the polymeric membrane, which would alter the absorbance measured in the test. 150 μL of enzyme loaded stomatocytes were mixed with 500 μL of CH_2Cl_2 for 30 mins. The final solution was then spin filtered with a centrifugal filter Unit 3 kDa (Millipore) to remove the organic solvent. The fraction collected was adjusted with buffer to the final volume of 150 μL. The measurements were performed in triplicate using 50 μL aliquots. For protein quantification, the Coomassie Plus (Bradford) assay kit was used (Pierce™) according to the manufacturer's instructions. In each cuvette both 1.5 mL of Coomassie reagent and 50 μL of sample were added. Before measuring the absorbance at 595 nm, all samples and the standard solutions were incubated for 5 mins at room temperature and the spectrophotometer was calibrated with a cuvette containing a blank solution.

Using the protein concentrations that were measured, the encapsulation efficiency (E.E. %) was determined by considering the protein concentration in the initial feed solution.

$$\text{E.E.}(\%) = \frac{\text{Protein concentration measured with the Bradford assay (mg mL - 1)}}{\text{Lowest value of protein concentration used in the feed (mg mL - 1)}} \times 100$$

References

1. L. K. E. A. Abdelmohsen, M. Nijemeisland, G. M. Pawar, G.-J. A. Janssen, R. J. M. Nolte, J. C. M. van Hest and D. A. Wilson, *ACS Nano*, 2016, **10**, 2652–2660.

1.3. Supplementary figures and table

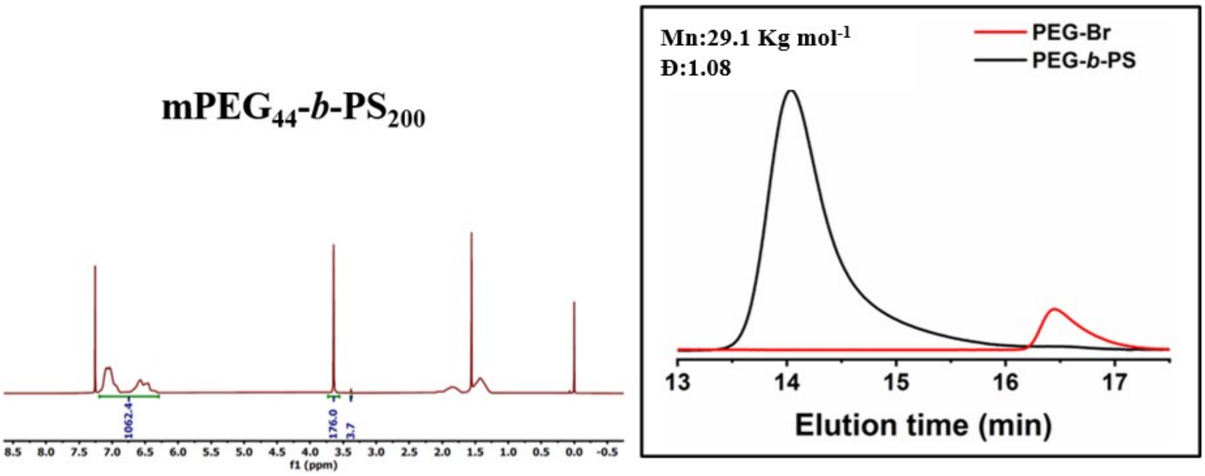

Figure S1-^{1}H-NMR and GPC spectra of block copolymer (PEG$_{44}$-*b*-PS$_{200}$) prepared by ATRP, Mn: 29.1 kg mol^{-1} and Đ:1.08.

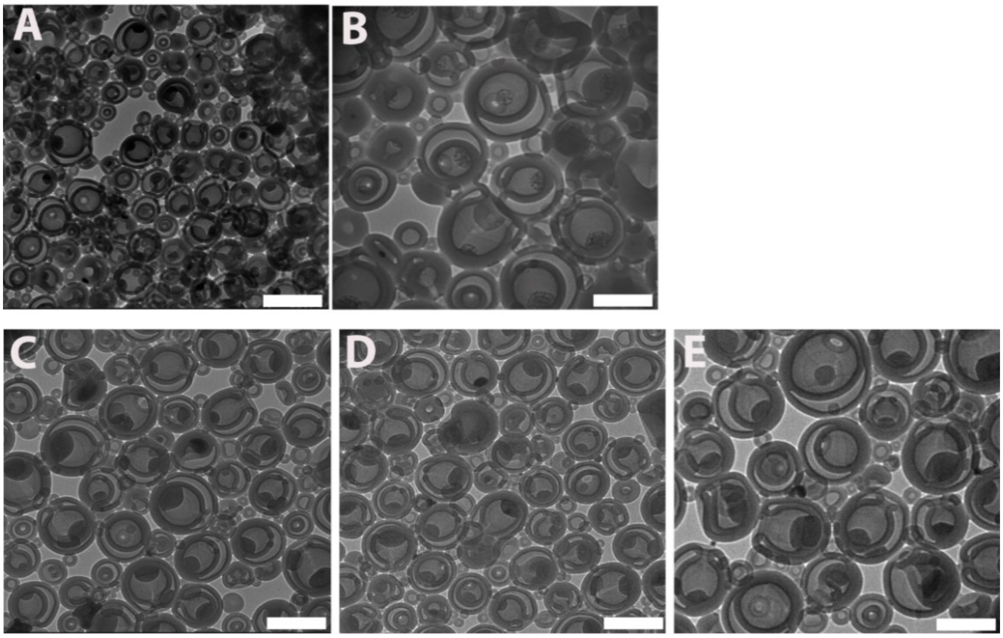

Figure S2-TEM pictures of **(A-B)**: NAL-*c*-CLE*n*A formed with 1%wt of genipin. Scalebars are 500nm and 200nm respectively. **(C-E)** combined 7α-HSDS/ 7β-HSDS *c*-CLE*n*As formed with 1%wt genipin. Scalebars are 500nm in C,D and 200nm in E.

TableS1- Optimisation of the formation of Neu5Ac using genipin cross-linked NAL-*c*-CLE*n*A in a microporous flow reactor. ME= Mixed Cellulose Ester; mPES= modified poly(ether sulfone).

(Top) Schematic setup and conversions to *N*-acetylneuraminic acid **3** with NAL-*c*-CLE*n*A- Conditions: A stock solution of *N*-acetyl-D-mannosamine, pH = 7 (**1**) and sodium pyruvate (**2**) (respectively 500 mM and 100 mM) in water (900 µL) was injected in a sample loop (1 mL) and pumped (at different flow rates) over the microporous hollow fibre reactor (mPES 10 kDa, 1.5 mL) loaded with genipin cross-linked NAL-*c*-CLE*n*A (0.34 g mL^{-1} of *c*-CLE*n*A, containing 18 mg of NAL) at various temperatures. The product flow was collected for at least 2.0 × t_R.

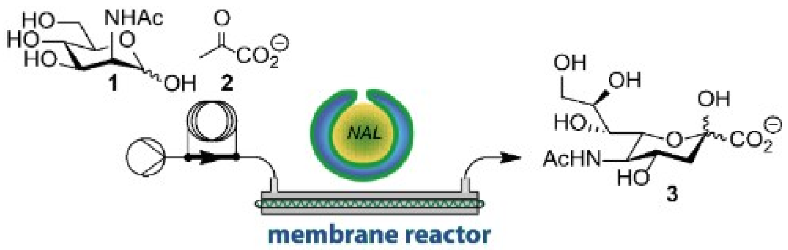

Entry	Flow (µLmin^{-1})	t_R (min)	T (°C)	Conversion (%)[a]	Membrane type
1	50	30	50	37	ME (0.1 µm, 1.5 mL)
2	25	60	50	41	
3	100	15	35	35	mPES (10kDa, 1.5 mL)
4	75	20	35	41	
5	50	30	35	47	
6	40	37.5	35	52	
7	30	50	35	60	
8	20	75	35	69	
9	20	75	50	50	
10	50	30	60	48	
11	50	30	70	22	
12	60	75	35	13	mPES (10kDa, 4.5 mL)
13	60	75	45	10	
14	75	60	50	16	

[a] The conversion toward Neu5Ac is measured by ^{1}H NMR spectroscopy of the crude reaction mixture.

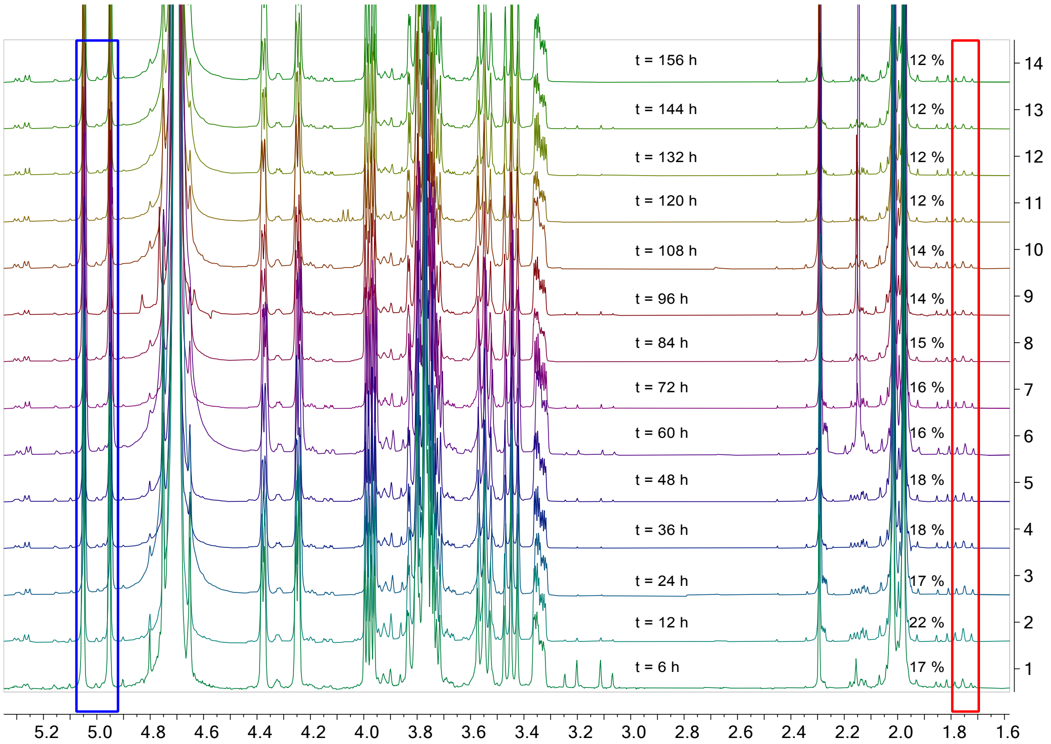

FigureS3- Stacked NMR plots of the continuous experiment described in Table S1. A gradual decrease in conversion of Neu5Ac is observed over time. Yields were determined using the integral sum of 5 distinct NMR signals: Blue box: H-1_{ax} and H-1_{eq} of *N*-acetyl-D-mannosamine. Red box: H-3_{ax} of *N*-acetylneuraminic acid

Table S2- Specific activity of 7α-HSDH and 7β-HSDH after cross-linking with different crosslinking agents. The initial activity of the purified enzymes was: 7α-HSDH 432.8 ± 18.5 U mg^{-1}; 7β-HSDH 2.38 ± 0.6. U mg^{-1}. Results are averages of triplicate maesurements performed on three different samples.

Glutaraldehyde= GA; Genipin= GP.

**[cross-linker]**	**7α-HSDH *c*-CLE*n*A U mg^{-1}**	**7β-HSDH *c*-CLE*n*A U mg^{-1}**
GP 1% wt.	382.66 ± 46.3	6.0 ± 1.4
GP 1.6 % wt	26.5 ± 2.2	nd
GP 0.75 % wt	16.8 ± 2.1	nd
GA 100mM	nd	nd

The difference in the activity observed could be explained by the effect of the changed ratio between enzyme concentration and cross-linker concentration ([enzyme:genipin]). When the amount of genipin was increased (1.6 wt%) the formation of cross-linked nano-aggregates was expected to occur faster but might have also caused deformation of the enzyme structure that resulted in a partial deactivation in case of 7α-HSDSH-*c*-CLE*n*A and in the total deactivation in the case of 7β-HSDSH. When less genipin was employed (0.75 wt%) the cross-linking rate was slower, which could have resulted in inefficient formation of nano-aggregates. In this case, 7β-HSDSH was completely deactivated while 7α-HSDSH-*c*-CLE*n*A maintained less than 5% of its native activity

Table S3-One-pot conversion using different dilutions (x10, x100,x1000) of the combined *c*-CLE*n*As and stomatocyte samples and different concentrations of NAD^+ (0.5 mM and 1mM). The conditions are given in 5.4.1.

**7α/7β-HSDH-stomatocytes** 202.2 µg/mL, 1 mM NAD^+				**7α/7β-HSDH-stomatocytes** 202.2 µg/mL, 0.5 mM NAD^+			
Time(min)	[hydroxysteroid] (mM)			Time(min)	[hydroxysteroid] (mM)		
	UDCA	7-oxo-LCA	CDCA		UDCA	7-oxo-LCA	CDCA
0	0.0	0.0	10.0	0	0.0	0.0	10.0
20	1.7	0.5	7.8	20	0.5	0.2	9.3
80	5.0	0.4	4.6	80	2.4	0,2	7.4
160	8.0	0.3	1.7	160	5.3	0.1	4.6
240	8.6	0.2	1.2	240	6.6	0.1	3.3
1440	9.0	0.3	0.7	1440	9.3	0.2	0.9

**7α/7β-HSDH-stomatocytes** 20.2 µg/mL, 1 mM NAD^+				**7α/7β-HSDH-stomatocytes** 20.2 µg/mL, 0.5 mM NAD^+			
Time(min)	[hydroxysteroid] (mM)			Time(min)	[hydroxysteroid] (mM)		
	UDCA	7-oxo-LCA	CDCA		UDCA	7-oxo-LCA	CDCA
0	0.0	0.0	10.0	0	0.0	0.0	10.0
20	0.1	0.5	9.4	20	0.0	0.1	9.8
80	0.4	0.4	9.2	80	0.1	0.1	9.8
160	0.9	0.4	8.7	160	0.3	0.1	9.6
240	1.4	0.4	8.2	240	0.5	0.1	9.4
1440	6.2	0.4	3.4	1440	2.8	0.2	7.0

**7α/7β-HSDH-stomatocytes** 2.0 µg/mL, 1 mM NAD^+				**7α/7β-HSDH-stomatocytes** 2.0 µg/mL, 0.5 mM NAD^+			
Time(min)	[hydroxysteroid] (mM)			Time(min)	[hydroxysteroid] (mM)		
	UDCA	7-oxo-LCA	CDCA		UDCA	7-oxo-LCA	CDCA
0	0.0	0.0	10.0	0	0.0	0.0	10.0
20	0.0	0.4	9.6	20	0.0	0.1	9.9
80	0.0	0.4	9.6	80	0.0	0.1	9.9
160	0.0	0.3	9.7	160	0.0	0.1	9.9
240	0.0	0.4	9.5	240	0.1	0.2	9.7
1440	0.3	0.4	9.3	1440	0.1	0.1	9.8

**7α/7β-HSDH-c-CLEnA**			
284.7 µg/mL, 1 mM NAD+			
Time(min)	[hydroxysteroid] (mM)		
	UDCA	7-oxo-LCA	CDCA
0	0.0	0.0	10.0
20	2.4	0.4	7.1
80	5.7	0.5	3.9
160	8.1	0.4	1.5
240	8.7	0.4	1.0
1440	9.0	0.3	0.7

**7α/7β-HSDH-c-CLEnA**			
284.7 µg/mL, 0.5 mM NAD+			
Time(min)	[hydroxysteroid] (mM)		
	UDCA	7-oxo-LCA	CDCA
0	0.0	0.0	10.0
20	0.5	0.1	9.4
80	2.8	0.2	7.0
160	4.8	0.2	5.0
240	5.7	0.2	4,2
1440	9.3	0.1	0,7

**7α/7β-HSDH-c-CLEnA**			
28.5 µg/mL, 1 mM NAD+			
Time(min)	[hydroxysteroid] (mM)		
	UDCA	7-oxo-LCA	CDCA
0	0.0	0.0	10.0
20	0.1	0.4	9.5
80	0.4	0.4	9.2
160	1.2	0.4	8.4
240	1.7	0.4	7.8
1440	7.2	0.3	2.5

**7α/7β-HSDH-c-CLEnA**			
28.5 µg/mL, 0.5 mM NAD+			
Time(min)	[hydroxysteroid] (mM)		
	UDCA	7-oxo-LCA	CDCA
0	0.0	0.0	10.0
20	0.1	0.2	9.8
80	0.1	0.2	9.7
160	0.4	0.1	9.5
240	0.6	0.1	9.3
1440	3.5	0.1	6.4

**7α/7β-HSDH-c-CLEnA**			
2.8 µg/mL, 1 mM NAD+			
Time(min)	[hydroxysteroid] (mM)		
	UDCA	7-oxo-LCA	CDCA
0	0.0	0.0	10,0
20	0.0	0.4	9.6
80	0.0	0.0	10.0
160	0.0	0.4	9.6
240	0.1	0.4	9.6
1440	0.5	0.4	9.1

**7α/7β-HSDH-c-CLEnA**			
2.8 µg/mL, 0.5 mM NAD+			
Time(min)	[hydroxysteroid] (mM)		
	UDCA	7-oxo-LCA	CDCA
0	0.0	0.0	10.0
20	0.0	0.1	9.9
80	0.0	0.1	9.9
160	0.0	0.1	9.9
240	0.0	0.2	9.8
1440	0.1	0.1	9.8

**7α-HSDSH-*c*-CLEnA + 7β-HSDH-*c*-CLEnA**			
2.6 µg/mL and 133 µg/mL respectively, 1 mM NAD+			
Time(min)	[hydroxysteroid] (mM)		
	UDCA	7-oxo-LCA	CDCA
0	0.0	0.0	10.0
60	4.0	0.4	5.6
120	5.4	0.4	4.2
200	6.2	0.3	3.5
1440	6.8	0.4	2.8

**7α-HSDSH-*c*-CLEnA + 7β-HSDH-*c*-CLEnA**			
2.6 µg/mL and 133 µg/mL respectively, 0.5 mM NAD+			
Time(min)	[hydroxysteroid] (mM)		
	UDCA	7-oxo-LCA	CDCA
0	0.0	0.0	10.0
60	0.9	0.1	8.9
120	1.1	0.2	8.7
200	1.4	0.1	8.5
1440	2.2	0.1	7.7

**7α/7β-HSDH-c-CLEnA**			
2847 µg/mL, 1 mM NAD+			
Time (min)	[hydroxysteroid] (mM)		
	UDCA	7-oxo-LCA	CDCA
0	0,0	0,0	10,0
60	9,0	0,3	0,8
1440	9,0	0,3	0,7
